# Clinicopathologic features and prognostic implications of NOK/STYK1 protein expression in non-small cell lung cancer

**DOI:** 10.1186/1471-2407-14-402

**Published:** 2014-06-04

**Authors:** Peng Chen, Wei-Miao Li, Qiang Lu, Jian Wang, Xiao-Long Yan, Zhi-Pei Zhang, Xiao-Fei Li

**Affiliations:** 1 Department of thoracic surgery, Tangdu hospital, Fourth Military Medical University, Xi’an, China

**Keywords:** NOK, Oncogene, Lung cancer, NSCLC, Survival, Prognosis, Immunohistochemistry

## Abstract

**Background:**

The expression of novel oncogenic kinase (NOK), a member of the protein tyrosine kinase (PTK) family, has been observed in several human malignancies including non-small cell lung cancer (NSCLC). However, the clinic relevance of NOK expression in NSCLC remains unclear.

**Methods:**

In this study, NOK expression in tumor cells was assessed using immunohistochemical methods in 191 patients with resected NSCLC. The association of NOK expression with clinicopathological parameters, including the Ki-67 labeling index (LI), was also evaluated. Kaplan-Meier survival analysis and Cox proportional hazards models were used to estimate the effect of NOK expression on survival.

**Results:**

Data showed that NOK was expressed in 75.4% and 14.1% of cancer lesions and corresponding adjacent non-cancerous tissue, respectively. Out of all the clinicopathological factors analyzed, NOK expression was significantly correlated with the grade of tumor differentiation (*P* = 0.035), pTNM stage (*P* = 0.020), lymphatic metastasis (*P* = 0.005) and high Ki-67 LI (*P* < 0.001). NOK positive NSCLC patients had a significantly shorter survival time (*P* = 0.004, Log-rank test) and the prognostic significance of NOK expression was apparent in squamous cell carcinoma patients (*P* = 0.022). Multivariate analysis indicated that NOK expression may be an independent prognostic factor in NSCLC (hazard ratio [HR], 1.731; *P* = 0.043).

**Conclusions:**

Our results indicate that NOK expression is of clinical significance and can serve as a prognostic biomarker in NSCLC.

## Background

Lung cancer is the leading cause of cancer-related death worldwide. Non-small cell lung cancer (NSCLC) accounts for most cases of lung cancer, however, the long-term survival rate of NSCLC patients remains unsatisfactory. A majority of NSCLC patients die from recurrent disease and distant metastases even after undergoing curative surgical resection [1-3]. There is an urgent need to identify new prognostic markers that can facilitate a better assessment of the survival probabilities and optimized therapies for individual patients.

Novel oncogenic kinase (NOK), also known as a putative serine/threonine and tyrosine receptor protein kinase (STYK) 1, was identified as a new member of the protein tyrosine kinase (PTK) family by Liu et al. [4,5]. It has a single putative transmembrane domain and an intracellular domain possessing tyrosine kinase activity but lacks an extracellular domain for binding specific ligands. Previous studies showed that NOK shares homology with members of the platelet-derived growth factor/fibroblast growth factor receptor superfamily and the overexpression of NOK in BaF3 cells induced tumorigenesis and metastasis in nude mice [5,6]. Furthermore, overexpression of NOK was detected in acute leukemia, ovarian cancer, breast cancer and lung cancer, but the prognostic role of NOK was not found [7-11]. A recent report also indicates NOK is functionally involved in Akt-glucose synthase kinase (GSK)-3β pathway, which is related with epithelial-to-mesenchymal transition (EMT) [12].

To study the clinicopathologic features and prognostic implications of NOK expression in patients with NSCLC, we investigated the expression of NOK in NSCLC by immunohistochemical staining and assessed the relationships between NOK expression and clinical parameters.

## Methods

### Patients and tissue samples

Paraffin-embedded tissue specimens from 191 patients with confirmed NSCLC, collected from 2007 to 2010, were analyzed from an archived thoracic oncology tissue repository at the Department of Thoracic Surgery of Tangdu Hospital. Patients who received preoperative chemotherapy, radiotherapy or epidermal growth factor receptor (EGFR)-targeted therapy were excluded from this study. Detailed information was obtained from the medical records of the enrolled patients in a computerized registry database including patient age, gender, smoking history, clinical manifestation, surgical method, tumor status, histological differentiation, nodal status and follow-up information. Follow-up lasted through 30 October, 2012, with a median follow-up period of 39 months for living patients (range, 23-64months). The day of surgery was considered as the starting day for estimating postoperative survival time. Histological classification of tumors was reviewed by pathologists and based on the World Health Organization criteria. All tumors were staged according to the pathological tumor/node/metastasis (pTNM) classification (7^th^ edition) of the International Union against Cancer [13]. The study protocol was approved by the Regional Ethics Committee for Clinical Research of the Fourth Military Medical University. All patients provided written informed consent for use of their medical records and tissue specimens for research purposes.

### Immunohistochemistry

Tissue blocks were cut into 5-μm sections and mounted on silane-coated slides. The slides were then dewaxed in xylene and rehydrated through a graded series of ethanol solution. Endogenous peroxidase activity was blocked by immersing the slides in a solution of 3% hydrogen peroxide in methanol for 30 min. Antigen retrieval was performed by microwaving sections in 10 mM citrate buffer (pH 6.0) at 95°C for 20 min. To reduce nonspecific binding, slides were blocked with goat serum for 30 min. Then, the sections were incubated in a humidified chamber at 4°C overnight with primary anti-NOK (diluted 1:100, Abcam, USA) or anti-Ki-67 (diluted 1:50, Thermo, USA) antibodies. After washing three times in PBS (phosphate-buffered saline), the slides were incubated for 60 min with a labeled polymer En Vision+. Peroxidase activity was visualized with the DAB Elite kit (Dako, Denmark), and the slides were counterstained with hematoxylin. To confirm the specificity of the immunostaining, negative controls were obtained by replacing the primary antibody with PBS.

### Evaluation of immunohistochemical staining

Five randomly fields from each section were viewed under a light microscope (Leica DM4000B, Germany) at × 400 magnification. The expression of NOK was scored by multiplication of the percentage of positive tumor cells and the staining intensity. Initially, the percentage of positive cells was scored as 0 (0%), 1 (1-10%), 2 (11-50%), and 3 (51-100%). Thereafter, intensity of staining was scored as follows: 0 (negative), 1 (weakly positive), 2 (moderately positive), and 3 (strongly positive). By ROC analyses, the case with a final scores ≥ 3 was classified as positive (Sensitivity 70.7, Specificity 90.6). For Ki-67, the expression of Ki-67 was assessed based on the labeling index (LI) determined by counting 500–1000 tumor cells randomly selected in a high-power field. The median value of positive tumor cells was 31% in the current series, therefore, we defined tumors with ≥31% of Ki-67 as high Ki-67.

All slides were assessed by 3 independent investigators who were blinded to the clinical features and outcomes. The final immunohistochemical staining score reported is the average of the scores from the three investigators.

### Statistical analysis

Associations between NOK expression and clinicopathological parameters were evaluated using the χ^2^-test. Survival was examined using the Kaplan-Meier method, and the significance of the difference was evaluated using the log-rank test. Correlation analyses of the survival time and various clinicopathological variables were performed by univariate and multivariate analyses using the Cox regression model. *P* < 0.05 were considered to be statistically significant. All analyses were performed with SPSS 18.0 software (SPSS, Inc., Chicago, IL).

## Results

### Patient characteristics

The clinicopathologic characteristics of the patients are summarized in Table 1. There were 37 female and 154 male patients with a median age of 61 years (range, 28–81 years). The patients were diagnosed with squamous cell carcinoma (SCC; n = 98, 51.3%) and adenocarcinoma (ADC; n = 93, 48.7%). Histopathologic diagnosis included: well differentiation-42 (22.0%), moderately differentiation-98 (51.3%), and poorly differentiation-51 (26.7%) tumors. Postoperative staging evaluation demonstrated stage I disease in 47 patients, stage II disease in 81 patients, stage III disease in 60 patients, and stage IV disease in 3 patients.

**Table 1:** Association between NOK expressions and clinicopathological features in NSCLC patients

**Variables**	**No. of cases**	**NOK expression**		
		**Positive (%)**	**Negative (%)**	*P*-value^*^
Sex				
Male	154	114 (74.0)	40 (26.0)	0.371
Female	37	30 (81.1)	7 (18.9)	
Age				
<60	90	70 (77.8)	20 (22.2)	0.470
≥60	101	74 (73.3)	27 (26.7)	
Smoking history				
Smoker	133	100	33	0.618
Non-smoker	58	42	16	
Differentiation				
Well + moderately	140	100 (71.4)	40 (28.6)	0.035
Poorly	51	44 (86.3)	7 (13.7)	
Histological type				
Squamous	98	71 (72.4)	27 (27.6)	0.332
Adenocarcinoma	93	73 (78.5)	20 (21.5)	
pT factor				
T_1–2_	148	110 (74.3)	38 (25.7)	0.525
T_3–4_	43	34 (79.1)	9 (20.0)	
pN factor				
N_0_	88	58 (65.9)	30 (34.1)	0.005
N_1–2_	103	86 (83.5)	17 (16.5)	
pTNM stage				
I-II	128	90 (70.3)	38 (29.7)	0.020
III-IV	63	54 (85.7)	9 (14.3)	
Ki-67 labeling index				
<31	94	57 (60.6)	37 (39.4)	<0.001
≥31	97	89 (91.8)	8 (8.2)	

*P-value of χ^2^-test are shown.

### Pattern of NOK expression in NSCLC and correlation with clinicopathological parameters

**Figure 1:**
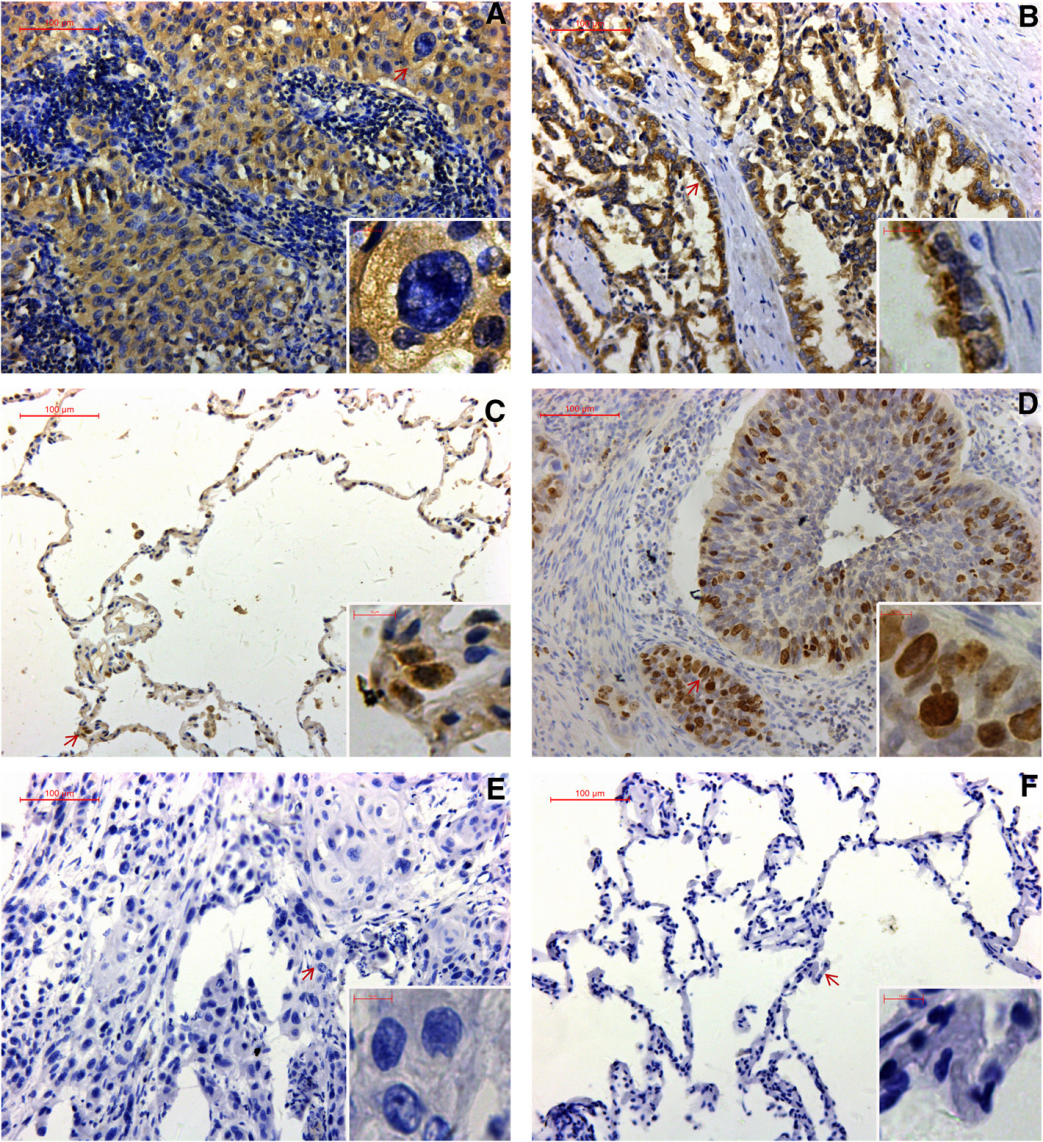
**Expression of NOK and Ki-67 in NSCLC. (A) **Positive NOK staining in SCC. **(B)** Positive NOK staining in ADC. **(C)** Positive NOK staining in corresponding adjacent non-cancerous tissue. **(D)** Positive Ki-67 staining in NSCLC. **(E)** Absence of NOK staining in NSCLC. **(F)** Absence of NOK staining in corresponding adjacent non-cancerous tissue.

Overall, 75.4% (144/191) tumor sections were classified as NOK positive while 14.1% (27/191) corresponding adjacent non-cancerous tissue sections were classified as NOK positive. Positive staining was mainly located in the cytoplasm (Figure 1).

In order to evaluate the role of NOK in NSCLC, we investigated whether NOK expression was associated with any of the clinicopathological variables (Table 1). The results showed that NOK expression was significantly associated with differentiation (*P* = 0.035), pN factor (*P* = 0.005), pTNM stage (*P* = 0.020) and Ki-67 labeling index (*P* < 0.001). No significant relationship was noted between NOK expression and sex (*P* = 0.371), age (*P* = 0.470), smoking history (*P* = 0.618), histological type (*P* = 0.332), and pT factor (*P* = 0.525).

### Relationship between NOK expression and survival in NSCLC patients

**Figure 2:**
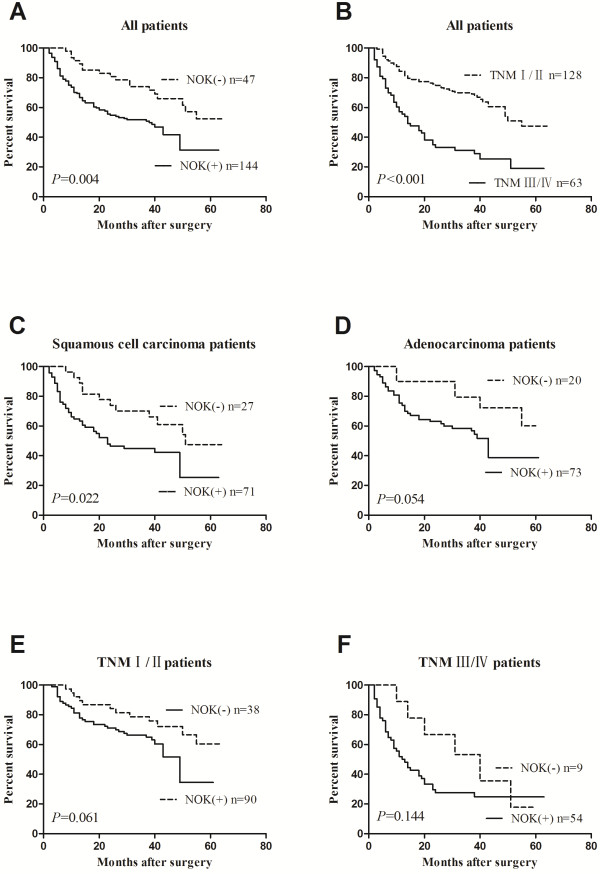
**Kaplan-Meier survival analysis for NSCLC patients.** The P-value was determined using the log-rank test. **(A)** Comparison of the overall survival (OS) between NOK negative and NOK positive NSCLC patients. **(B)** Comparison of the OS between pTNM I/II and pTNM III/IV patients. **(C)** Comparison of the OS between NOK negative and NOK positive SCC patients. **(D)** Comparison of the OS between NOK negative and NOK positive ADC patients. **(E)** Comparison of the OS between NOK negative and NOK positive pTNM I/II patients. **(F)** Comparison of the OS between NOK negative and NOK positive pTNM III/IV patients.

To investigate the relationship between NOK expression and the clinical outcome of NSCLC patients, the correlation between patient survival and NOK expression status was analyzed. Patients with NOK positive expression had a significantly worse prognosis than those with NOK negative expression (*P* = 0.004, Figure 2A). The median survival time of patients with NOK-positive NSCLC (n = 144) was 38 months (95% confidence interval [CI] = 26-50 months) and the mean survival time was 34 months (95% CI = 30-39 months), whereas the median survival time of patients with NOK-negative NSCLC (n = 47) had not yet been reached and the mean survival time was 48 months (95% CI = 43-54 months). Among other factors such as patient age, sex, smoking history, tumor histological type, grade of tumor differentiation and pTNM stage, only the pTNM stage and grade of tumor differentiation were significantly associated with patient survival. The median survival time of patients with pTNM I/II tumors (n = 128) was 55 months (95% CI was not reached), whereas the median survival time of those with TNM III/IV tumors (n = 63) was 14 months (95% CI = 7–21 months; *P* < 0.001, Figure 2B). Patients with well or moderately differentiated tumors (n = 140, median survival time was not reached) had a longer survival time than those with poorly differentiated tumors (n = 51, median survival time = 19 months, 95% CI = 11-27 months; *P* < 0.001).

Then, the patients were divided into the SCC and ADC groups. The median survival time of patients with NOK positive SCC (n = 71) was 23 months (95% CI = 12-34 months), whereas the median survival time of those with NOK negative SCC (n = 27) was 51 months (95% CI was not reached). SCC patients with NOK positive expression had a significantly worse prognosis than those with NOK negative expression (*P* = 0.022, Figure 2C). By contrast, the median and mean survival time of patients with NOK-positive ADC (n = 73) was 43 months (95% CI = 35-50 months) and 37 months (95% CI = 30-43 months), respectively, whereas the mean survival time of those with NOK-negative ADC (n = 20) was 50 months (95% CI = 42-57 months). There were no significant difference in the survival rate with respect to NOK expression among ADC patients (*P* = 0.054; Figure 2D).

In order to investigate the relationship between NOK expression and the clinical outcome of NSCLC patients according to the different stage, we divided patients into the pTNM I/II and pTNM III/IV groups. Among patients with I/II-stage tumors, the median and mean survival time of patients with NOK-positive tumors (n = 90) was 49 months (95% CI = 41-57 months) and 40 months (95% CI = 34-45 months), respectively, whereas the mean survival time of those with NOK-negative tumors (n = 38) was 51 months (95% CI = 45-57 months). There were no significant difference in the survival rate with respect to NOK expression (*P* = 0.061, Figure 2E). Among patients with III/IV-stage tumors, the median survival time of NOK positive patients (n = 54) was 12 months (95% CI = 7-17 months), whereas that of NOK negative patients (n = 9) was 40 months (95% CI = 17-63 months). There were no significant difference in the survival rate with respect to NOK expression (*P* = 0.144, Figure 2F).

To further assess whether NOK expression represents a prognostic parameter in patients with NSCLC, regression analysis using the Cox’s proportional hazards model was performed. The covariate parameters included several clinicopathological factors in addition to NOK, as shown in Table 2. In univariate analysis, factors including NOK expression, pTNM stage and grade of differentiation showed a significantly higher hazard ratio for a poor prognosis. Moreover, multivariate analysis was carried out using the significant factors observed in univariate analysis. The results showed that, besides the TNM stage and grade of differentiation, NOK expression was an independent prognostic factor (*P* = 0.043, Table 2). These results strongly indicated that the NOK expression in NSCLC patients is closely related to a poor prognosis.

**Table 2:** Cox proportional hazards model analysis of variables affecting survival in NSCLC patients

**Variables**	**Categories**	**Univariate analysis**		**Multivariate analysis**	
		**HR (95% CI)**	***P*-value**	**HR (95% CI)**	***P*-value**
Age (years)	≥60/<60	1.330 (0.88-2.01)	0.178		
Sex	Male/female	0.835 (0.51-1.37)	0.473		
Smoking history	Smoking/Non-smoking	0.887 (0.58-1.37)	0.588		
Histological type	Squamous/adenocarcinoma	1.341 (0.89-2.02)	0.160		
Grade of differentiation	Poor/well + moderate	2.578 (1.70-3.90)	<0.001	1.814 (1.19-2.78)	0.006
pTNM stage	I/II/ III/IV	2.331 (1.79-3.05)	<0.001	2.132 (1.61-2.82)	<0.001
NOK expression	Positive/negative	2.121 (1.25-3.59)	0.005	1.731 (1.02-2.94)	0.043

HR, hazard ratio; CI, confidence interval.

## Discussion

PTKs are important regulators of intracellular signal-transduction pathways mediating development and multicellular communication in metazoans. Their activity is normally tightly controlled and regulated. In contrast, alterations in the expression of various PTKs, in their activation, and in the signaling molecules lying downstream of the receptors play important roles in the development of cancer [14,15]. PTK expression has been associated with poor prognosis and is a predictor of metastasis in NSCLCs [16]. Some PTKs, such as EGFR and human epidermal growth factor receptor (HER)2, play important roles in lung cancer and have emerged as targets of new drug therapies [17-19]. As a novel member of the PTK family, the clinical relevance of NOK with NSCLC warrants evaluation.

In this study, we performed immunohistochemical analysis of surgically resected NSCLC specimens to investigate NOK expression in clinical samples. NOK protein was found to be mainly localized in the cytoplasm. Immunohistochemical analysis revealed that 75.4% (144/191) of the tumor sections analyzed were positive for NOK protein expression, while 14.1% (27/191) of corresponding adjacent non-cancerous tissue were NOK-positive. In contrast to our results, Amachika T et al. reported NOK mRNA was detected in 97.6% (40/41) of lung cancer tissues [8]. The differences in the detection methods and sample sizes might account for these differences. However, both the previous study and our analyses showed that NOK expression in cancerous tissues is significantly higher, compared to that in non-cancerous tissues. These data suggest that NOK may be a new diagnostic tool or biomarker for lung cancer. Subsequently, we found that NOK expression in NSCLC was notably associated with some clinical parameters, such as the pN factor and pTNM stage. These findings indicate that NOK may play an import role in the metastasis of NSCLC. Recently, Li J et al. reported that NOK forms complexes with both Akt and GSK-3β and that this complex formation enhanced the phosphorylation of GSK-3β [12]. Indeed, phosphorylated-GSK-3β expression is frequently associated with EMT (epithelial-mesenchymal transition), which promotes the dissemination of cancer cells from primary tumors [20-22]. Our results are consistent with those of the report from Li J. et al. [12], in that they support a role for NOK in promoting metastases of NSCLC.

This study is the first, to our knowledge, to indicate an association between NOK-positivity in NSCLC patients and poor prognosis and to suggest that NOK expression may be an independent prognostic factor of NSCLC patients. Furthermore, the prognostic significance of NOK expression was apparent in SCC patients, while the difference in the survival rate with respect to NOK expression among ADC patients was not significant. Further studies, in a larger cohort of patients with lung ADC, are needed to determine whether NOK expression is a predictor of poor prognosis in lung ADC patients. We also investigated whether NOK expression was associated with prognosis in early stage NSCLC patients. The results did not show apparent prognostic significance of NOK expression in early stage patients as well as in late stage patients. The basis of the relationship between NOK expression and poor prognosis needs further evaluation. We hypothesize that NOK expression may be related to cancer cell proliferation and metastasis. Liu L et al. reported that NOK-expressing tumor cells could promptly invade and spread to various organs and form metastatic foci, eventually leading to the rapid death of animals [5]. Li YH et al. reported that NOK is involved in the activation of the intracellular RAS/ mitogen-activated protein kinase (MAPK) signaling pathway [23]. MAPK signaling pathways have been implicated in the pathogenesis of a variety of human cancers, including lung cancer [24,25]. Our previous studies indicated that NOK gene expression could improve the proliferation and invasion capabilities of human lung adenocarcinoma cells [26,27]. The results of the current study also demonstrate a probable relationship between NOK expression and a high Ki-67 LI in NSCLC patients. All of the above mentioned data may, in part, account for the association between NOK expression and poor prognosis in NSCLC.

There were some shortcomings present in this study. Due to the limitations of sample size and follow-up, the median survival time in many groups could not be obtained. To respond the patient's survival, we listed the mean survival time, which was failed to accurately respond the patient’s survival as the median survival time. In addition, this study did not investigate the relationship between the expression level of NOK gene and the pathogenesis of lung cancer, which was very important for a biomarker validation. To compensate for these shortcomings, we intend to carry out further multi-center clinical studies, expand the sample size and enrich the means of detection.

Recently, EGFR- tyrosine kinase inhibitors (TKIs) and ALK inhibitors have been used widely in clinical settings as standard cancer therapies, and PTK-targeted therapies have demonstrated clinical success in the treatment of NSCLC [28,29]. Furthermore, a study by Ding X et al. showed that NOK was co-localized with EGFR in endosomes and that it participates in a post-internalization step of EGFR [30]. Taken together with the results in this study, we believe that NOK might be a potential drug therapy target for NSCLC patients. However, the mechanism underlying the promotion of NSCLC by NOK needs to be examined further.

## Conclusions

We showed that a high proportion of NSCLCs express NOK and that NOK expression is significantly associated with grade of tumor differentiation, pTNM stage, and lymphatic metastasis. Moreover, our study is the first to indicate a role for NOK as an independent factor predictive of poor prognosis in NSCLCs.

## Abbreviations

NSCLC: Non-small cell lung cancer; NOK: Novel oncogenic kinase; STYK: Serine/threonine and tyrosine receptor protein kianse; PTK: Protein tyrosine kinase; GSK: Glucose synthase kinase; EGFR: Epidermal growth factor receptor; LI: Labeling index; SCC: Squamous cell carcinoma; ADC: Adenocarcinoma; CI: Confidence interval; HR: Hazard ratio; HER: Human epidermal growth factor receptor; MAPK: Mitogen-activated protein kinase; TKIs: Tyrosine kinase inhibitors.

## Competing interests

The authors declare that they have no competing interests.

## Authors’ contributions

PC, WML, QL and XFL contributed to the study concept and design. WML, JW, and ZPZ acquired the data. QL, XLY and PC analyzed and interpreted the data. PC and WML drafted the manuscript. XFL, QL and XLY critically revised the manuscript for important intellectual content. PC and XLY performed the statistical analysis. All authors have read and approved the final manuscript. Supervision was done by XFL and ZPZ.

## Pre-publication history

The pre-publication history for this paper can be accessed here:

http://www.biomedcentral.com/1471-2407/14/402/prepub
